# The Activities of the Slovenian Strategic Council for Nutrition 2023/24 to Improve the Health of the Slovenian Population and the Sustainability of Food: A Narrative Review

**DOI:** 10.3390/nu15204390

**Published:** 2023-10-16

**Authors:** Zlatko Fras, Boštjan Jakše, Samo Kreft, Žiga Malek, Tanja Kamin, Nika Tavčar, Nataša Fidler Mis

**Affiliations:** 1 Division of Medicine, Centre for Preventive Cardiology, University Medical Centre Ljubljana, 1000 Ljubljana, Slovenia; zlatko.fras@kclj.si; 2 Faculty of Medicine, University of Ljubljana, 1000 Ljubljana, Slovenia; 3 Independent Researcher, 1000 Ljubljana, Slovenia; bj7899@student.uni-lj.si; 4 Faculty of Pharmacy, University of Ljubljana, 1000 Ljubljana, Slovenia; samo.kreft@ffa.uni-lj.si; 5 Institute for Environmental Studies (IVM), Vrije Universiteit Amsterdam, 1081 HV Amsterdam, The Netherlands; ziga.malek@vu.nl; 6 Faculty of Social Sciences, University of Ljubljana, 1000 Ljubljana, Slovenia; tanja.kamin@fdv.uni-lj.si; 7 Umanotera, The Slovenian Foundation for Sustainable Development, 1000 Ljubljana, Slovenia; nika@umanotera.org; 8 Ministry of Health, 1000 Ljubljana, Slovenia; 9 Division of Paediatrics, University Medical Centre Ljubljana, 1000 Ljubljana, Slovenia

**Keywords:** healthy nutrition, sustainable nutrition, dietary guidelines, chronic diseases, monitoring, healthy lifestyle

## Abstract

The health status of individuals in Slovenia across age groups is a matter of concern, as current unsustainable lifestyle choices are already leading to various chronic noncommunicable diseases (NCDs). Outdated national dietary guidelines, their inconsistent implementation, and a lack of structural changes represent obstacles to promoting healthy and sustainable nutrition. Limited access to and rising prices of healthy, sustainable foods, in addition to the high availability of low-priced, highly processed foods, increase the risk of NCDs. The lack of systematic health monitoring and early disease detection poses a challenge. Global and local environmental issues, resistance, and/or the inability to adopt healthier diets hinder individuals from changing their nutritional behaviours. In this narrative review, we provide an overview of the current situation in Slovenia as well as planned activities initiated by the Slovenian government and the Prime Minister’s Strategic Council for Nutrition, aiming to make progress in supporting healthy and sustainable nutrition, limiting food waste, and increasing the availability of healthier foods for all. Improving the sustainability of the Slovenian food system can contribute to several Sustainable Development Goals (SDGs), ensuring Slovenia’s commitment to internationally agreed-upon targets. This could lead Slovenia to take a role as a pilot country in testing and implementing the necessary systemic changes, which could be further applied in other countries.

## 1. Introduction

Approximately one billion people worldwide have inadequate access to food, while many more consume substandard diets that contribute to the development of diet-, lifestyle-, and environment-related chronic noncommunicable diseases (NCDs), such as obesity, diabetes, cardiovascular disease, and cancer [1]. Chronic NCDs are among the leading causes of death and premature mortality [2]. Due to their prolonged course and often severe disabilities, they also represent a considerable social burden [3,4].

In parallel to the human health crisis, the global food system is a primary driver of biodiversity loss [5] and a leading cause of climate and nature crises, with 34% of anthropogenic greenhouse gas (GHG) emissions derived from food production, ranging from 21% to approximately 37% [6,7]. A recent study employing a model–data integration approach revealed that 57% are attributed to the production of animal-based food [8]. NCDs are the leading cause of mortality and health care costs worldwide. The burden of NCDs is constantly increasing [9,10]. NCDs contributed to as many as 73% of all deaths in 2017. The total number of deaths caused by NCDs increased by 23% from 2007 to 2017, representing an additional 7.61 million annual deaths in 2017 versus 2007. Additionally, the estimated years of life lost (YLLs) increased for ischaemic heart disease (ranked first in 2017) and stroke (ranked third) [11]. Some consider NCDs to be diseases of the modern age due to the overall increase in life expectancy. Although people live longer, older individuals often live with disabilities and chronic diseases [12]. While there has been considerable progress in the clinical management of NCDs, there is a need for more effective preventive strategies [13].

The economic burden of unhealthy lifestyles, which can lead to chronic diseases, can no longer be endured or further ignored, especially as most NCDs are preventable [14,15,16]. Based on a systematic review of 28 studies, it is evident that the financial burden imposed by four NCDs—cardiovascular disease (CVD), cancer, diabetes, and chronic respiratory disease—within the European Union (EU) is substantial. This burden accounts for a minimum of 25% of the total health expenditure, resulting in an economic loss equivalent to nearly 2% of the EU’s gross domestic product (GDP). Some data even suggest that NCD-related costs could be as high as 70–80% of the total healthcare expenditure [17]. In 2018, across the EU-28 member countries, cardiovascular disease alone was responsible for 1.8 million deaths, which translates to a loss of 1.5 million potential years of productivity. This lost productivity was estimated to cost approximately EUR 37 billion [18]. In Slovenia, CVD ranked as the second most significant contributor to premature mortality in 2017, accounting for nearly 88,000 YLLs (29%) [19]. In Europe (EU-27, Iceland, Norway, Switzerland, and the United Kingdom), the number of newly diagnosed cancer cases has steadily risen, experiencing an approximately 50% increase from 2.1 million to 3.1 million cases between 1995 and 2018. A recent study reported a staggering 250 million disability-adjusted life years (DALYs) attributed to cancer in 2019 [20].

Furthermore, in 2015, the economic impact of cardiovascular diseases on Slovenia was estimated to be as high as EUR 800 million. Considering that CVD accounts for only 21% of all DALYs attributed to NCDs, the total economic burden of NCDs across the EU could potentially reach up to EUR 4 billion [19], representing approximately 7% of the GDP [21]. Moreover, in Slovenia, cancer takes the top spot as the leading cause of premature mortality measured in YLLs. Among various cancer types, colorectal cancer stands out as a significant contributor to premature mortality, accounting for nearly 5% of all YLLs (14,214 YLLs) in 2017. Interestingly, while premature mortality due to cancer has been on a consistent rise among men over the past two decades, there has been a decline in this trend for women since 2004 [19]. In Slovenia, the comprehensive economic impact of cancer, which includes health expenditures on cancer care, informal care costs, and productivity losses due to premature mortality and morbidity, totaled approximately EUR 600 million in 2018 [22].

To successfully address and prevent the causes of these diseases, a strong emphasis must be placed on lifestyle medicine [23]. It is crucial to prioritise a healthy lifestyle as a key strategy to promote and maintain as many healthy years as possible across all age groups. Overwhelming evidence indicates that modifiable lifestyle factors, including unhealthy dietary habits, insufficient physical activity, excessive alcohol consumption, tobacco use, illegal drug use, and psychosocial factors (such as chronic stress, lack of social support, and community), play a major role in the pathogenesis and incidence of NCDs such as CVD, various types of cancer, metabolic syndrome, obesity, diabetes, chronic lung diseases, and musculoskeletal disorders. Modifiable lifestyle factors impact both overall life expectancy and the incidence of chronic diseases [23,24,25,26,27,28]. Unhealthy nutrition and insufficient physical activity are closely associated with the occurrence and maintenance of biological risk factors for coronary heart disease (CHD), including elevated blood pressure and cholesterol levels, as well as several other highly prevalent NCDs [29,30,31,32,33]. A study conducted by British researchers estimated that consistently following current dietary guidelines would lead to a one-third reduction in the risk of CVD among healthy middle-aged and older men and women by lowering blood pressure and lipid levels [34]. Healthy and sustainable diets are characterised by appropriate energy intake and a diverse range of plant-based foods, limited consumption of animal-based foods, preference for unsaturated fats over saturated fats, and minimal consumption of refined grains, highly processed foods, added sugars, and salt. Shifting from current diets to healthy and sustainable diets can have important benefits for human health, potentially preventing 10.8–11.6 million deaths annually, which represents a reduction of 19–24% [1].

In Slovenia, the Prime Minister established the Strategic Council for Nutrition, one of several strategic councils that bring together experts from specific fields not typically involved in the executive branches of politics. This council serves as a consultative body, offering recommendations to improve various aspects related to nutrition and nutritional systems. These recommendations encompass a wide range of topics, including the health of various social and demographic groups, agriculture, the environment, infrastructure, and logistical challenges within the nutrition system, education, taxation of unhealthy foods, and subsidies for health-promoting food groups. Notably, the Strategic Council for Nutrition includes members from various ministries responsible for (a) health; (b) labour, family, social affairs, and equal opportunities; (c) education, science, and sport; (d) agriculture, forestry, and food; (e) environment, climate, and energy; and (d) digital transformation. In addition to members from these ministries, the Council includes members from the Biotechnical Faculty, Faculty of Medicine, Faculty of Social Sciences, Faculty of Pharmacy, University Medical Centre Ljubljana, Umanotera, the Slovenian Foundation for Sustainable Development, members from the private sector engaged in research on the impact of human nutrition on various health aspects, representatives for the promotion of local self-sufficiency, and members of the National Institute of Public Health of Slovenia (NIPH), a central national institution dedicated to studying, protecting, and improving the health of the population of Slovenia [35]. This inclusion aims to enhance interdepartmental ministerial coordination regarding the initiatives, proposals, and positions presented by the Strategic Council for Nutrition. Without such collaboration, achieving measurable effects through the Prime Minister’s consultative body over time would be challenging. We are primarily focused on revising healthy eating guidelines for children, students, and the general adult population. This comprehensive effort considers nutritional, cultural, environmental, socioeconomic, and communication factors. Furthermore, our pilot projects involve renovating elementary school canteens, establishing central school kitchens for smaller places with limited capacities as an option, and enhancing dietitian standards in primary schools. Additionally, we address food waste and promote plant-based meals in public institutions as an option with unchanged patterns of existing meals. We collaborate with the Ministry of the Environment, Climate, and Energy to reduce the environmental impact of food production and support digital transformation in the agriculture and food sectors. Our ultimate aim is to advance the sustainability of the food system in Slovenia through innovative approaches and practices.

The Strategic Council for Nutrition, with its diverse and inter/independent composition of experts [35], unanimously endorses the “Planetary Health Diet,” a universal reference diet developed by an international group of experts known as the EAT-Lancet Commission [1]. The EAT-Lancet Commission recommends (1) at least five servings of fruits and vegetables per day, not including potatoes; (2) carbohydrates primarily from whole grains, with low intake of refined grains and sugar contributing below 5% of total energy; (3) protein sources primarily from plants (soy foods, other legumes, and nuts), with occasional consumption of fish or other sources of omega-3 fatty acids and optional modest consumption of poultry and eggs, while red meat, especially processed meat, should be consumed in low or no amounts; (4) fat should come predominantly from unsaturated plant sources, with a low intake of saturated fats and no consumption of partly hydrogenated trans fats; and (5) moderate dairy consumption as an option [1]. The growing prevalence of chronic NCDs, along with their environmental consequences, including global warming, air, water, and soil pollution, and land use changes associated with deforestation, present a dual challenge that necessitates a transition towards nutrition that is both of higher quality and sustainable [1,36,37]. Dietary changes towards a healthy and sustainable diet are not only necessary [38,39,40,41] but also feasible [1,42,43] and have already been implemented to various degrees in many countries [44,45,46,47,48,49].

The aim of this narrative review was to assess scientific studies that have evaluated the populational burden of two key groups of NCDs in Slovenia, CVD and cancer, as well as the prevalence of unhealthy lifestyles and other major risk factors in Slovenia, NCD prevention projects in Slovenia, starting points for future changes and further improvement, and the role of the Slovenian Strategic Council for Nutrition and its working groups. Based on the findings, the Working Groups of the Slovenian Strategic Council for Nutrition for 2023/2024 will reflect on suitable measures and interventions considering points i–viii. Scientific studies published between 2010 and 2023, primarily in the PubMed/Medline, Scopus, and Web of Science databases, were identified using specific search terms for nutrition, disease, obesity, and lifestyle. Additionally, some conference proceedings and reports related to Slovenian data were included in the search. Publications that did not specifically quantify our search topics were excluded. This resulted in a total of 151 articles selected for this review. The studies reviewed included human clinical trials.

## 2. The Populational Burden of Two Key Groups of NCDs in Slovenia: CVD and Cancer

In Slovenia, the main behavioural risk factors for death are nutritional risks (i.e., poor diet), tobacco smoking, and alcohol consumption. Dietary risks, including low fruit and vegetable intake and high sugar and salt consumption, were implicated in 16% of all deaths in 2019, which is approximately the same as the EU average. Tobacco smoking contributed to an estimated 15% of all deaths in Slovenia, while approximately 5% were attributable to alcohol consumption and 1% to low physical activity. The research (data from the European Social Survey 2014), which included four lifestyle data points (physical activity, fruit and vegetable intake, alcohol consumption, and smoking) from 20 European countries in a large sample (n = 34,993), showed that, on average, only 5.8% of Europeans reported a healthy lifestyle. Slovenians, with 4.5% of adults reporting a healthy lifestyle, ranked fifth worst [50]. Moreover, mortality rates from preventable causes exceeded the EU average, while mortality rates from treatable causes were lower than the EU average [51]. Notably, life expectancy at birth has improved by 5.3 years, from 76.1 years in 2000 to 81.3 years in 2019 (females live approximately 7.6 years longer), and is in line with the EU-28 average [52]. Moreover, healthy life expectancy at birth has also improved by 4.2 years, from 66.5 years in 2000 to 70.7 years in 2019 [53]. Currently, according to data on life expectancy (for 2021), female healthy life expectancy is longer than that of males (67.3 years versus 63.7 years) [54]. Finally, standardised death rates and the percentage of different groups of disease causes for Slovenia are presented in Table 1, along with a comparison to the EU average (both overall and separated into EU members before and after 2004) [55].

### 2.1. Cardiovascular Diseases

Although mortality rates have significantly decreased since 2000, CVDs remain the leading cause of death in Slovenia. In 2021, they accounted for 33% of all deaths (38% among women and 27% among men). This means that of the approximately 20,000 deaths that occur every year in Slovenia, approximately 7500 are caused by CVDs. Ischaemic heart disease is responsible for one in ten deaths, with a similar mortality rate reported for stroke. These groups of diseases also pose a major burden in terms of disability, hospitalisation rate, and drug consumption [56].

Despite the rapid increase in average life expectancy in Slovenia over the last two decades (by approximately 5 years for both men and women), there has been a rise in morbidity and mortality associated with behavioural and biological risk factors. The data from the recent European Health Interview Survey shown in Table 2 demonstrate that regarding the prevalence of (self-reported) behavioural CVD risk factors, Slovenia is significantly lagging behind the more advanced European states [57,58,59]. On the other hand, due to substantial variations in definitions, assessment methodologies, and the population groups included, the prevalence rates of hypercholesterolemia and arterial hypertension in Slovenia can be presented only as estimates. Based on self-report studies, approximately 28% of individuals aged 25–74 years reported hypercholesterolemia, while 24% reported arterial hypertension [60]. Importantly, the sensitivity of self-reporting compared to direct measurement is estimated to be 51% [61]. This suggests that the actual prevalence of hypercholesterolemia or arterial hypertension may be approximately twice as high as that reported in self-reported studies. Therefore, it is possible that up to 56% of Slovenian adults have hypercholesterolemia, and approximately 48% have arterial hypertension. However, according to the Registry of Participants in the National Programme of Primary Prevention of Cardiovascular Diseases, which collected data between 2002 and 2017 from 1,102,021 adult individuals aged 35–70 years, 66% had total cholesterol levels higher than 5 mmol/L, and 33% had blood pressure levels higher than 140/90 mmHg [62]. Notably, in the age group included in this programme, the prevalence of hypercholesterolemia might have been overestimated and the prevalence of arterial hypertension might have been underestimated. The global prevalence of hypercholesterolemia and arterial hypertension in adults in 2019 was estimated at 32% for females and 34% for males aged 30–79 years [63].

Two opportunistic cross-sectional studies conducted in 2018 and 2019, which involved one blood pressure measurement during “Arterial Hypertension Day” in various locations across Slovenia, estimated the prevalence of hypertension to be approximately twice as high (58% and 61%, respectively) [64,65]. However, the sample was not representative, particularly due to the low response rate and the mean age of the participants (56 years), suggesting that the prevalence could have been overestimated. Nonetheless, the upward trend of arterial hypertension prevalence is concerning due to its association with a greater risk of various cardiovascular manifestations, chronic kidney disease, dementia, erectile dysfunction, and other conditions [66,67,68,69,70].

### 2.2. Cancer

In recent years, Slovenia has experienced an annual diagnosis rate of cancer of approximately 14,000 individuals, with over 6000 deaths. Notably, more than 94,000 individuals currently live with a cancer diagnosis. The burden of cancer in Slovenia is primarily dominated by the five most common cancer sites: skin, colon and rectum, lung, breast, and prostate. These five cancer sites account for nearly 60% of all new cases. Moreover, while the incidence of common cancers has been increasing at an average rate of 3.0% per year, the incidence of rare cancers has remained stable. It is important to consider that Slovenia is facing an ageing population, which suggests that the burden of cancer will continue to rise even if risk factors remain unchanged. Although only mortality from pancreatic cancer increased during the last two decades among the ten leading causes of death, lung cancer mortality is increasing among women, and lung cancer remains the most frequent cause of death by cancer among Slovenians [71].

Encouragingly, there has been an improvement in the survival rate of Slovenian cancer patients over the past two decades. The five-year net survival increased by 11%, with greater growth observed among men. Age and stage at diagnosis continue to play important roles in patient survival. Notably, there have been considerable advancements in the survival rates for common cancers such as melanoma, colorectal cancer, and lung cancer for both sexes. Progress has also been made in the two most prevalent sex-specific cancers: breast cancer in women and prostate cancer in men. However, it is worth mentioning that the substantial progress in prostate cancer survival may be influenced by lead-time bias due to the indiscriminate use of prostate-specific antigen (PSA) testing during the study period, which likely artificially prolonged survival [72]. Collectively, these studies emphasise the importance of continuous monitoring of the cancer burden, improvements in cancer care, and the development of evidence-based preventive (lifestyle) strategies to address the challenges posed by cancer in Slovenia.

Evidence shows that a poor diet, a lack of exercise, being overweight, and excessive alcohol consumption all increase the risk of developing certain cancers. However, by maintaining a healthy weight, engaging in regular exercise, and making healthy food choices, we can significantly reduce this risk [73,74].

### 2.3. NCD Prevention Projects in Slovenia

In 2015, Slovenia adopted the Resolution on the National Programme on Nutrition and Physical Activity for Health 2015–2025, aiming to build upon the achievements of previous national programmes (2005–2010) and the National Programme for the Promotion of Physical Activity for Health Improvement. The objective is to enhance and further develop well-designed activities to align with the collective efforts of the European Union in mitigating the rise in obesity and chronic NCDs. The Slovenian Strategic Council for Nutrition plays a crucial role in advocating for improved integration of health with environmental considerations and sustainability in future initiatives [75]. A 2023–2025 action plan is currently under development to implement the 2015–2025 National Programme on Nutrition and Physical Activity for Health. This plan aligns with the Strategic Council for Nutrition’s efforts and will undergo a public review before finalising interdepartmental coordination.

In addition, the NIPH has developed an integrated chronic disease prevention programme, part of the Together for Health programme [76], over the past decades. Primary health care services under the statutory health insurance system in Slovenia offer various services to adults, including workshops on healthy lifestyle changes in areas such as nutrition, physical activity, mental health, smoking, alcohol consumption, obesity, and diabetes at health promotion centres [76]. Approximately 50,000 users attend these workshops annually [77]. One of the major challenges is achieving a high response rate in the population. Despite the rise in chronic diseases and unhealthy lifestyles, particularly among those with a lower socioeconomic status [76,77,78], only 14% of the population has attended at least one workshop, even though 48% are aware of the Together for Health programme services [76]. A research project is currently underway to understand the factors that influence people’s responses to and participation in the programme and to identify differences between individual factors (e.g., awareness of and intention to participate in a prevention programme, attitudes towards prevention, motives, health concerns, the symbolic meaning of health and health care, willingness to change behaviour) and systemic factors (e.g., social norms, accessibility of programmes). In addition to formal health promotion programmes led by health agencies such as the NIPH, the Slovenian Ministry of Health is instrumental in funding several nongovernmental programmes and other programmes that promote and support healthier behavioural choices related to nutrition and physical activity [79].

## 3. Prevalence of Unhealthy Lifestyles and Other Major Risk Factors in Slovenia (by Age Group)

### 3.1. Nutritional Status (i.e., Anthropometrical Status and Dietary Intake) of Slovenian Children and Adolescents

The prevalence of overweight and obesity among Slovenian children and adolescents has experienced a substantial increase over the past three decades [80]. The percentage of children classified as obese tripled for both sexes between 1989 and the late 2000s, while the percentage of overweight children doubled [81]. Specifically, the prevalence of overweight increased from 11.8 to 18.9% among boys and from 11.4 to 17% among girls. Additionally, the rate of obesity rose from 1.9 to 5.9% among boys and from 1.7 to 4.7% among girls, suggesting that overall, both overweight and obesity rates increased more among boys than among girls [81]. Furthermore, it is concerning that between 1989 and 2019, the amount of subcutaneous fat (rather than muscle mass) increased in children of all age groups and sexes [80]. However, the decreased prevalence of childhood obesity observed after 2010 was primarily attributed to a loss of lean mass rather than fat mass, as indicated by additional findings [81]. Importantly, the data regarding these worrisome anthropometric parameters in children and adolescents cannot be solely attributed to insufficient physical activity, given that the Slovenian child population was previously regarded as one of the most physically active worldwide before the recent coronavirus disease 2019 (COVID-19) pandemic [82]. Research has revealed that 86% of boys and 79% of girls meet the physical activity frequency recommendations set by the World Health Organisation (WHO) [83]. However, starting in the 2010/2011 school year, a programme called Healthy Lifestyle was introduced in approximately 30% of Slovenian primary schools. This programme added 2 extra hours of physical education per week, taught by physical education teachers, to the existing 2 or 3 h. While participation was not mandatory and not all children in each school took part, the programme managed to involve approximately 20% of all 7–14-year-old children in Slovenia by 2015. The Healthy Lifestyle initiative aims to promote physical activity for better health. It is free of charge and open to everyone, with a special focus on children who are not active in sports outside school. In the same year, Slovenia also implemented the School Meals Act and National Dietary Guidelines 2010. These guidelines ensured healthy school meals and removed vending machines from all Slovenian schools. The researchers reported that the interventions implemented thus far have been effective, but there is also a suggestion that their long-term effects may be diminishing [81]. The COVID-19 pandemic exacerbated the decline in lean mass among children, leading to a considerable deterioration in physical fitness and health-related anthropometric parameters. During the 2019/2020 school year, there was an unprecedented increase in the proportion of overweight children. The pandemic resulted in an almost 17% increase in obesity among boys and an 18% increase among girls. Both boys and girls experienced a significant increase in the prevalence of morbid obesity, with an 80% increase among boys and a 60% increase among girls [80,84].

The Slovenian nationally representative dietary survey, SI. Menu 2017/2018, provided evidence that the nutritional intake of vitamin D, fibre, folate, iron, and vitamin B12 among adolescents (specifically, those aged between 10 and 17 years) did not meet the recommended levels. The intake of these nutrients was deficient in adolescents of both sexes at a 100% rate for vitamin D, 91% for fibre, 88% for folate, 44% for iron, and 38% for vitamin B12. Among girls, the deficiency in iron and vitamin B12 was even more pronounced, at 73% and 44%, respectively [85,86,87,88]. Another study focusing on adolescents aged 14–16 years in Slovenia also highlighted worrisome nutritional trends. The findings revealed that the majority of adolescents fell short of consuming the recommended amounts of fruits, vegetables, fish, milk, and cereals. Conversely, they tended to exceed the recommended intake of meat and meat products by 320%, sweets and savoury snacks by 453%, and sodium by 200–300 times. These trends underscore the importance of addressing and improving adolescent nutrition in Slovenia [89]. Additionally, an analysis of the chemical and nutritional composition of lunches served in Slovenian educational institutions, such as kindergartens and primary schools, revealed that these meals are inadequately balanced according to modern recommendations. They lack sufficient energy, fibre, polyunsaturated fats, and various micronutrients while surpassing the recommended upper limits of saturated and total fats and salt [90,91,92,93]. These findings indicate a systemic issue with the inadequate and inconsistent implementation of contemporary dietary guidelines.

It is crucial to recognise that the responsibility for inadequate diets and nutrient intake among children in educational institutions cannot be solely attributed to the nutrition provided by these institutions. Nutrition in the home environment, including meals during afternoons, weekends, and holidays, is beyond the control of educational institutions, especially given that children in the upper grades of primary and secondary school do not eat any meals at school. Importantly, the current state of inadequate dietary patterns and nutrient intake in educational institutions may stem from various multidimensional factors associated with modern living. These factors encompass various obstacles that hinder children from adopting healthy eating habits within the family. These include a fast-paced lifestyle, limited free time on weekdays [94], societal norms that promote unhealthy lifestyles [95], extensive promotion of unhealthy food to children through media channels [96,97], and subsidised food and products, including high-fat meat and dairy items, which may increase the risk of chronic diseases [98,99,100,101]. Collectively, these factors contribute to the intricate landscape of dietary habits and nutrition-related challenges experienced by children in educational settings.

### 3.2. Nutritional (i.e., Anthropometrical Status and Dietary Intake) and Health Status of Slovenian Adults and Older Adults

According to a nationally representative cross-sectional survey (SI. Menu 2017/2018) involving 780 Slovenian adults aged 18 to 64 years and older adults aged 65 to 74 years, the prevalence of overweight and obesity was 59% (68% of males and 50% of females) and 74% (79% of males and 73% of females), respectively [87,102]. More recently, during the post-COVID-19 pandemic period, researchers conducted a study among 844 adults aged 18 to 81 years and estimated that 43% were either overweight or obese (42% of adults (53% of males and 35% of females) and 64% of older adults (78% of males and 55% of females)) [103].

In addition, a substantial proportion of the adult population (including older adults) exhibited inadequate intake levels of key nutrients: 100% (100%) for vitamin D, 90% (84%) for fibre, and 88% (88%) for folate. Additionally, 32% of all adults and 46% of older adults had insufficient intake of iron and vitamin B12, with an alarming 76% of females aged 18–50 years being affected [85,86,87,88]. In terms of dietary intake compared with food-based dietary guidelines, their typical diets are unbalanced due to high amounts of consumed meat and meat products, foods high in sugar, fat, and salt, and low intake of fruits and vegetables and milk and dairy products [104]. These findings highlight the widespread inadequacy of nutrient intake among Slovenian adults, mirroring the patterns observed in younger age groups [89,105].

## 4. Physical Activity as an Inseparable Part of Diet and a Healthy Lifestyle

Globally, 7% of all deaths and 8% of deaths related to heart and vascular diseases are attributed to physical inactivity [31]. A study conducted in 2013 across 142 countries estimated that, conservatively, physical inactivity alone posed a burden of over EUR 50 billion on health care systems [9]. Among EU member countries in 2018, physical inactivity resulted in annual economic costs of approximately EUR 80 billion [106]. Research in the United States revealed that each inactive individual costs the health care system approximately EUR 1440 more annually compared to an active person [107]. If the prevalence of physical inactivity worldwide does not decrease, it is projected that by 2030, it will lead to approximately 500 million new cases of common NCDs [108]. Underlining its significance, physical inactivity ranks as the 10th leading risk factor for the most common diseases [29]. Regular physical activity, whether integrated into daily life or through organised exercise, is associated with at least a 20–30% reduced risk of more than 25 prevalent chronic NCDs and premature death [30]. The greatest health benefits are derived from a combination of aerobic physical activity (such as walking, cycling, and swimming) and strength training (using body weight, exercise equipment, or a fitness setting) [109]. Regular, sufficient, and appropriate physical activity also plays a crucial role in controlling and balancing energy intake [110], which is particularly important with today’s lifestyles, in which individuals often regain part of the excess body weight they lose between holidays or breaks (due to overconsumption of high-calorie foods) [111]. Frequent and regular physical activity is closely linked to recommendations for maintaining a healthy body weight and composition. Studies among children, adolescents, and adults consistently show that the best results are achieved through a combination of appropriate physical activity/exercise and a healthy diet, especially when it is part of a healthy and active lifestyle [112,113,114,115,116].

The recommendations for regular activity for healthy adults from the Slovenian Strategic Council for Nutrition include the following:

- Any form of physical activity is better than physical inactivity, and it is never too late to start.
- On weekdays, individuals should aim for 5000 to 10,000 brisk (moderate-intensity) steps on varied terrain (e.g., nearby hills) daily.
- On weekends, individuals should aim for 7000 to 12,000 brisk (moderate-intensity) steps on varied terrain (e.g., nearby hills) daily.
- Strength training should be performed at least three times a week, lasting 30–45 min per session.
- Proper nutrition, hydration, safety, and good company during physical activity and in general should be ensured.
- Physical activity should be adapted to age, health condition, physical capabilities, and goals.
- Individuals should focus on activities they enjoy; if they do not like walking, they might enjoy swimming, dancing, playing tennis, etc.

## 5. Starting Points for Future Changes and Further Improvements

Slovenia’s future dietary guidelines will aim to promote healthy eating and overall nutritional well-being, originating from the dietary and culinary practices of our ancestors, who practised much more sustainable food production and consumption. We need to keep in mind natural resources for sustainable food production within Slovenia, including growing more organic foods while also reducing the environmental impact. The achievement of sustainable climate protection, water and soil conservation, and the preservation and restoration of biodiversity requires collective action and joint efforts. Slovenia is therefore committed to various international agreements and initiatives related to climate change, biodiversity protection, and sustainable development. As a signatory to the Paris Agreement on climate change, the country has pledged to reduce greenhouse gas (GHG) emissions. While agriculture contributes only 10% to total national GHG emissions [117], the emissions of Slovenia’s food systems are potentially much higher if accounting for GHG emissions outside Slovenia’s borders. Slovenia imports a considerable part of the food consumed within its borders, mostly processed food but also 16% of consumed meat, 52% of fruits and vegetables, and a variety of agricultural inputs, such as feed for livestock [118]. Moreover, a diet predominantly relying on animal products, such as in the case of Slovenia, has demonstrably higher GHG emissions [119]. In a recent study, it was found that Slovenians lack sustainable dietary habits or attitudes regarding health, the environment, animal welfare, and consideration for dietary minorities [120].

In addition to reducing GHG emissions, Slovenia aims to reduce the overall impacts on the environment through the European Green Deal, initiated by the European Commission in 2019. The Green Deal places a strong emphasis on promoting sustainable practices that will reduce impacts on water and soil resources and reverse biodiversity loss [121]. This is particularly important for Slovenia, which mostly imports food from other European Union member states, where agriculture is among the main drivers of habitat and species degradation [122]. The Farm to Fork Strategy, within the European Green Deal, aims to transform food systems to be healthy, environmentally friendly, and fair [123,124]. This strategy recognises the importance of transitioning to healthy diets from sustainable food systems. To foster healthier and more environmentally friendly eating habits, it is crucial to prioritise the consumption of locally sourced food. Whenever feasible, choosing seasonal and organically produced options while avoiding highly processed foods that are high in sugar, salt, saturated fats, and preservatives is recommended. Additionally, minimising food waste and reducing packaging are integral aspects. By promoting and implementing these healthier and planet-friendly meal options through national and local public policies as well as educational institutions, we can contribute to the creation of more sustainable food systems [125]. Improving the sustainability of the Slovenian diet can contribute to different Sustainable Development Goals (SDGs) (although undoubtedly on a smaller scale due to Slovenia’s size), therefore ensuring Slovenia’s commitment to internationally agreed-upon targets: SDG 6 (clean water and sanitation) through decreased pollution and use of freshwater resources; SDG 12 (responsible consumption and production) through decreasing food waste; SDG 13 (climate action) through decreased GHG emissions; and SDG 15 (life on land) through smaller impacts on soil and biodiversity [126]. Furthermore, Slovenia’s proactive endeavours in this domain, despite its status as one of the smaller EU countries, present an opportunity for the nation to assume a pioneering role in implementing and testing essential systemic changes towards a more sustainable future. This successful model could subsequently serve as a blueprint for other countries or regions to adopt and follow.

In addition, adhering to nutritional and health recommendations has positive environmental impacts [49,127]. We will focus on utilising our natural resources for sustainable food production in Slovenia, including the cultivation of organic foods, while striving to minimise our environmental footprint. Improving the sustainability of the food system can therefore lead to synergies with health for Slovenia’s citizens. In line with these commitments and goals, Slovenia has taken initiatives to promote “healthy and sustainable nutrition” with dedicated support from the current government. This underscores the country’s commitment to aligning its actions with climate commitments and sustainable development objectives.

Importantly, achieving sustainable nutrition for the broader community requires an integrated and transdisciplinary approach. Cultural, social, physiological, geographical, and economic factors present various challenges, making a transition to a strictly plant-based diet unfeasible for everyone [128]. Initiating changes for a healthier future can begin with implementing many more healthy and sustainable eating practices. This includes shifting towards a higher ratio of plant protein to animal-based protein intake, predominantly but not exclusively through plant-based eating. This would involve reducing the consumption of animal-based foods, especially red meat and meat products, as well as ultra-processed foods with unfavourable nutritional compositions. Simultaneously, it would involve increasing the intake and diversity of plant-based foods such as vegetables, legumes, whole grains, fruits, and nuts/seeds [129]. Adopting such practices can have a dual beneficial impact: reducing the risk of common NCDs globally and simultaneously improving the environment, which is essential [1,130,131,132]. Recent government campaigns have sometimes promoted less healthy and unsustainable foods. The ministry contemplates a “green tax reform” to solve this issue. The Ministry of Finance, the Ministry of Agriculture, and the Ministry of Health aim to update food policies to promote sustainable and eco-friendly diets, aligning with climate and environmental concerns. The policies should align with the goal of the Strategic Food Council, which is to provide affordable, safe, and high-quality food while minimising negative impacts on health, the environment, and the climate. To address these complex issues, the government is taking a comprehensive approach, including targeted subsidies, excise duties, value-added tax adjustments, and budget-funded advertising campaigns. The Ministry of Health and the National Institute of Public Health have presented various fiscal policies, and the WHO has recommended interventions for taxing unhealthy foods, with excise duties being a common approach (Slovenian document that cites, among others, several WHO documents) [133,134,135,136]. However, categorising foods based on their health content is challenging due to value-added tax regulations. Resistance is expected from the food industry, which sells highly processed foods, and possibly from a portion of the public as well. Clear communication is crucial to conveying the government’s aim to encourage healthier and more sustainable eating habits, especially among financially vulnerable groups. To navigate these complexities, we propose creating a multidisciplinary working group within the Strategic Council for Nutrition and the Strategic Council for Healthcare. These groups will comprise experts in nutrition, nutrition systems, and fiscal matters from the Ministry of Finance. They will consider feedback from the Strategic Council for Nutrition and the Strategic Council for Healthcare members, study international best practices, and develop final recommendations.

We recognise that providing accurate and comprehensive dietary information and influencing empowerment processes would help people make healthier dietary choices and gradually lead them to more sustainable lifestyles. In addition to informing and empowering individuals, cross-sectoral cooperation and various structural changes need to be made in the direction of promoting healthier eating habits in environments where people spend a great deal of time, such as schools and workplace canteens, and where they buy food to prepare meals at home. Healthy foods need to be tasty and more readily available and affordable than less healthy alternatives.

## 6. Slovenian Strategic Council for Nutrition and Its Working Groups

In December 2022, the Slovenian prime minister instituted the Slovenian Strategic Council for Nutrition as an advisory entity to the prime minister. The council’s primary objective is to develop proposals for measures aimed at ensuring accessible, safe, and high-quality food with minimal adverse effects on human health, the environment, and the climate. Additionally, the council will provide recommendations and expert guidance for the revision of national dietary guidelines; the reduction in food waste; setting a goal for increased production of organic foods accessible to all; raising public awareness regarding the necessity of dietary habit changes for health, environmental, and climate-related reasons; and undertaking other designated tasks [137].

Based on the findings, the Working Groups of the Slovenian Strategic Council for Nutrition for 2023/2024 will reflect on suitable measures and interventions considering the following (points i–viii, Table 3):

## 7. Conclusions

We conclude that promoting a healthy lifestyle, including healthy eating and overall nutritional well-being, along with increased access to locally and sustainably produced organic foods and food waste prevention, is crucial for lowering the risk of common NCDs in Slovenia. Scientific evidence supports the important role of a healthy and sustainable dietary pattern in overall health.

An interdisciplinary approach is essential to effectively integrating more sustainable, healthy diets into a complex system of food production and supply. To achieve that objective, European dietetic associations and the European dietitian workforce are dedicated to promoting healthier and more sustainable dietary patterns, emphasising affordable diets that are diversified, nutritious, and less resource-intensive and generate minimal waste. The European Federation of the Associations of Dietitians (EFAD) urges European countries to review their national food-based dietary guidelines to incorporate sustainability aspects as a unifying force for health and the environment. Moreover, EFAD calls upon policymakers, civil society, the food industry, farmers, and consumers to actively support actions and policies that facilitate transitions towards a healthier and greener Europe [151]. Such collaborative actions are essential for promoting a holistic approach to nutrition and sustainable food systems, benefiting both human well-being and the environment.

However, several challenges and obstacles need to be addressed. The Slovenian government, together with the Slovenian Strategic Council for Nutrition, is actively working towards achieving its goal. The Slovenian government leads by, for example, promoting healthy and sustainable meals in their canteens and aims to empower individuals and organisations to adopt positive dietary changes. Structural adjustments can be more effective when aligned with broader international commitments, although they may not ensure a seamless transition. Slovenia is resolute in improving nutrition policies, programmes, and educational resources to promote a healthier and more sustainable diet for all. Transitioning from current diets to healthier and more sustainable choices will substantially benefit the health of Slovenian citizens and the overall environment.

## Figures and Tables

**Table 1:** Standardised death rates and % calculated shares of different groups of disease causes for Slovenia compared to the European averages [55].

Year		SDR, All Causes	SDR, Major NCD 30–69 *	% of All Deaths	SDR, Circulatory Diseases	% of All Deaths	SDR, Neoplasms	% of All Deaths
2000	Slovenia	799	402	50.3	315	39.4	204	25.5
	WHO European region	946	529	55.9	458	48.4	176	18.6
	EU members	737	381	51.7	311	42.2	189	25.6
	EU members before 2004	656	323	49.2	245	37.3	183	27.9
	EU members after 2004	1013	592	58.4	537	53.0	210	20.7
2010	Slovenia	600	289	48.1	218	36.3	196	32.7
	WHO European region	786	422	53.7	363	46.2	158	20.1
	EU members	595	297	49.9	218	36.6	168	28.2
	EU members before 2004	530	248	46.8	165	31.1	161	30.4
	EU members after 2004	836	478	57.2	416	49.8	193	23.1
2018	Slovenia	531	244	45.9	180	33.9	184	34.6
	WHO European region	669	355	53.1	280	41.8	145	21.7
	EU members	546	260	47.6	174	31.9	154	28.2
	EU members before 2004	495	220	44.4	135	27.3	147	29.7
	EU members after 2004	745	408	54.8	328	44.0	181	24.3

SDR: standardised death rate; NCD: noncommunicable diseases; * major NCD 30–69: circulatory + cancer + DM (diabetes mellitus) + respiratory disease in adults aged 30–69 years.

**Table 2:** Prevalence of various BMI values, the amount of leisure-time health-enhancing physical activity, and the amount of daily fruit and vegetable consumption in Slovenia, its neighbouring countries, and EU-27 countries (in % of population over 15 years of age) [57,58,59].

	BMI [59]				HEPA [58]			Fruit and Vegetable Consumption [57]		
	Underweight	Normal Weight	Overweight	Obese	Zero min/wk	1–149 min/wk	≥150 min/wk	Zero Portions/Day	1–4 Portions/Day	≥5 Portions/Day
Austria	2.6	46.3	34.4	16.7	25.1	24.6	21	31.8	61.1	7.2
Croatia	1.7	34.5	41.2	22.6	58.6	22	8.8	27.5	65.5	7.0
Hungary	2.7	38.9	34.5	23.9	43.4	27.9	11.8	33.1	56.8	10.1
Italy	3.9	51.4	33.2	11.4	65	16.8	9.4	23.0	65.1	11.8
Slovenia	1.6	41.8	37.3	19.4	39.1	23.0	15.5	27.0	65.5	7.5
EU-27	2.9	45.8	35.2	16.0	49.8	20.2	13.9	36	52.1	11.9

BMI: body mass index (kg/m^2^); HEPA: health-enhancing physical activity (min/wk).

**Table 3:** Measures and interventions that the Working Groups of the Slovenian Strategic Council for Nutrition proposed to the prime minister for consideration in 2023/2024.

(i)	In collaboration with the National Institute for Public Health, the Extended Professional College for Paediatrics, and the Nutrition and Dietetics Association, we will conduct a health review and develop new healthy eating guidelines for children and adolescents in educational institutions, as well as for students. Led by the Ministry of Labour, Family, Social Affairs, and Equal Opportunities, we will initiate the renovation of the student nutrition system.
(ii)	Led by the Ministry of Health, we will write new “planetary health” and sustainable eating guidelines for the general adult population in Slovenia (i.e., the Slovenian food plate). We will consider nutritional and health implications, Slovenian culinary traditions, cultural contexts, environmental factors (i.e., carbon and water footprint, water and soil health, food loss and waste, biodiversity), and socioeconomic (accounting for the most vulnerable populations) and communication elements.
(iii)	Pilot projects led by the Ministry of Education will focus on the renovation of dining halls, the establishment of regional central school kitchens and school ecogardens, improving national standards for dietitians in primary schools, and integrating the food catalogue of the Chamber of Commerce of Slovenia.
(iv)	Led by the Ministry of Agriculture, Forestry, and Food and the relevant stakeholders, we will tackle food waste, working together with the public and private sectors to better find solutions to deal with food waste and food waste prevention. The main activities are within the EU Platform on Food Losses and Food Waste [138,139].
(v)	To encourage and promote public sector leadership in healthy and sustainable diets by supporting public institutions in changing their menu to be healthier and more sustainable, the benefits of plant-based menu options in public institutions were reviewed, considering food appreciation, food waste reduction, feasibility surveys, health and environmental calculations, and examples of existing practices in Slovenia (the canteen of the Slovenian government, some kindergartens, and schools), Portugal [47], Italy [42,140,141], the UK [142], Finland [143], Spain [144], Sweden [43], the USA [145,146,147,148,149,150], and Brazil [46]). It will be necessary for the government to incentivise innovation and to invest in a healthy and sustainable food system, including reforming standards for buying more fresh, seasonal, sustainable foods from local farmers and implementing a mandatory system to support public institutional canteens in raising standards towards healthy and sustainable options.
(vi)	The Slovenian Strategic Council for Nutrition works closely with the Ministry of the Environment, Climate, and Energy, which provides financial support for several of the aforementioned activities. Their vision aims to contribute to the transformation of Slovenian agriculture into a more regenerative, resilient, sufficient, and efficient system, to increase the area under organic agriculture and reduce food’s environmental impacts throughout the entire food supply chain, from producers to consumers. One of their initiatives is leading a pilot project to establish a methodology for calculating the carbon footprint of menus in public schools, funding cooking workshops for school cooks and nutritionists, and providing a range of educational materials and recipes; the overarching goal is to improve various aspects of Slovenia’s food sustainability index.
(vii)	Led by the Ministry of Digital Transformation, the digital transformation of the agricultural and food sectors will be undertaken, intending to achieve sustainable and resilient food systems and the digitisation of food procurement and meal preparation processes in public institutions.
(viii)	The monthly meetings of the Working Groups of the Slovenian Strategic Council for Nutrition incorporate diverse examples of successful global and local dietary practices in educational institutions, including proposing specific solutions to ensure the availability of healthy plant-based menu options alongside the existing balanced mixed meals; implementing such changes requires structural and personnel modifications to support this expanded choice.

## Data Availability

Not applicable.
